# Collecting Essential Data on Healthcare Professionals’ Career Trajectories With Life History Calendars

**DOI:** 10.3389/ijph.2026.1609261

**Published:** 2026-02-10

**Authors:** André Berchtold, Leonard Roth, Annie Oulevey Bachmann, Jonathan Jubin, Ingrid Gilles, Isabelle Peytremann-Bridevaux

**Affiliations:** 1 Institute of Social Sciences and Centre LIVES, University of Lausanne, Lausanne, Switzerland; 2 Department of Epidemiology and Health Systems, Centre for Primary Care and Public Health (Unisanté), University of Lausanne, Lausanne, Switzerland; 3 La Source School of Nursing, HES-SO University of Applied Sciences and Arts Western Switzerland, Lausanne, Switzerland; 4 Human Resources Direction, Lausanne University Hospital, Lausanne, Switzerland

**Keywords:** survey methodology, retrospective data collection, longitudinal studies, career trajectories, healthcare workforce, life history calendar

The study of career trajectories requires data that goes well beyond the different jobs a person has held during their lifetime. Numerous studies have shown that it is also necessary to take into account educational and professional background, including continuous education and detailed information on employments, as well as various life events related to family, illnesses, or external circumstances such as pandemics, or economic shocks [1, 2]. This is particularly true for the healthcare workforce (HCWF), where working conditions are difficult, staff shortages and turnover are problematic, with many career changes and relocations, people leaving their professions, or, conversely, entering the career later in life after initial training in another field [3–7].

To analyze complexity in healthcare career trajectories, it is suitable to have detailed longitudinal data covering many areas (professional, educational, family, health, etc.) and spanning several decades, ideally from childhood to the present. Longitudinal data can be collected either prospectively, over time, or retrospectively [8]. Prospective data is considered the gold standard [9], but it is costly to collect, subject to attrition, and, above all, it takes several years before data covering a sufficiently long period is finally available for valid analysis. The accelerated longitudinal design method [10] makes it possible to partially overcome this constraint by surveying people of all ages during only a few time points, then combining the responses of the different respondents to reconstruct complete pseudo-trajectories. However, this method completely ignores the cohort effect, i.e., the fact that, for example, the conditions for entering the labor market are very different today than they were 20 or 30 years ago. This means that accelerated longitudinal design is only suitable for situations that are very homogeneous over time, which is not the case for the study of healthcare professionals’ career trajectories. Another source of longitudinal data could be long-term longitudinal surveys, such as the Swiss Household Panel [11] or the German Socio-Economic Panel [12], which provide immediate access to long series of longitudinal data. However, these surveys pursue general objectives of understanding the population as a whole and therefore do not include sufficiently detailed data to study specific topics such as the HCWF.

The limitations of prospective longitudinal data motivated us to consider methods allowing data to be collected retrospectively. There are distinct advantages to such a retrospective approach, as all data up to the present moment can be collected at once, which significantly reduces collection costs and results in immediately available and complete data. However, retrospective data are often considered less reliable than prospective data, as they suffer from the cognitive limitations of human beings and their difficulty in accurately and comprehensively recalling events that may have happened decades ago [13]. This is particularly true when data are collected using a traditional questionnaire consisting of multiple questions. To overcome this, a special tool called the Life History Calendar (LHC) has been developed to improve the quality of retrospective data by maximizing the use of different cognitive processes related to memory and recollection [14].

**FIGURE 1:**
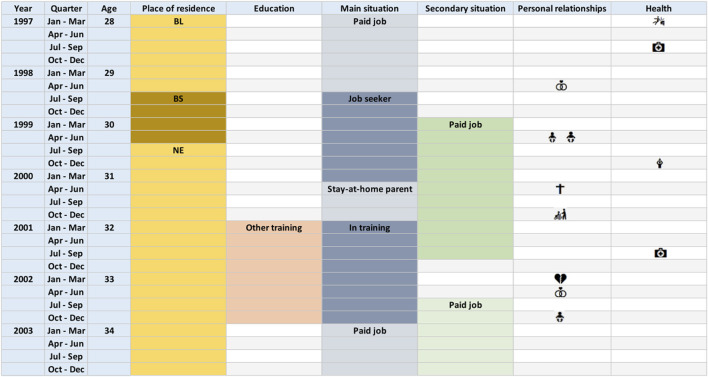
Example for the main screen of a Life History Calendar. Each row represents a trimester and each column, a different life domain. Periods are represented by colored blocks and specific events by icons (Switzerland, 2025).

An LHC, as shown in Figure 1, is a graphical tool in the form of a grid with rows representing periods of time (a month, a trimester, etc.) and columns each representing an area of life (education, health, family life, work, etc.) [15]. The various events that have occurred in each area of life, whether they were one-off events (e.g., the birth of a child) or of varying duration (e.g., training or employment), can be indicated in the corresponding columns, with additional details provided if required (exact qualification obtained at the end of training, employment rate, etc.). The decisive advantage of the LHC over other retrospective methods is that it allows the various events that have occurred in different areas to be visually linked, which helps to stimulate respondents’ memories by exploiting the cognitive processes associated with the distance in time from past events, their succession, and their positioning within larger periods [16, 17]. The quality of the data obtained can be further improved by inserting a number of cues into the calendar, such as the respondent’s age or a list of events known to many (presidential election, Olympic Games, etc.) [18, 19]. All of this improves both the number of events reported and their temporal accuracy.

LHCs can be used in a wide range of different fields, either to supplement prospective longitudinal surveys or as the sole data collection tool. LHCs have often been filled out on paper with the help of an interviewer, but it is also possible to use online versions of this tool that can be completed independently [20, 21]. Research shows that the different versions of LHCs provide reliable and comprehensive retrospective data [22]. However, LHCs also have certain limitations. These include the difficulty of distinguishing between missing information and events that genuinely did not occur, the need for careful selection of temporal and contextual cues to support recall without biasing responses, and, in self-administered survey environments, the particularly high demands placed on the clarity and precision of the instructions provided, as respondents must rely exclusively on written guidance to complete the calendar.

**FIGURE 2:**
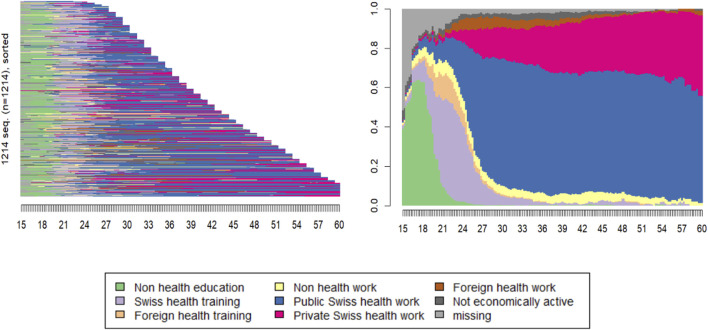
Example of a preliminary sequence analysis exploring 1214 career trajectories of Swiss healthcare professionals. The categorical states at the bottom are constructed based on information collected through a Life History Calendar. The x-axes correspond to the age (in years) of the individuals. The left plot shows all trajectories sorted by length, with the end corresponding to the individual’s age at time of data collection. The right plot shows the state distribution at each time point (Switzerland, 2025).

Sequence analysis [23, 24] is the most common statistical method for studying life trajectories such as those collected using an LHC (Figure 2). Beyond simply listing and representing the trajectories observed for an individual in each area of life, this method makes it possible to identify the most typical sequences, relate the sequences observed in each area, divide individuals into a finite number of groups, interpret these groups using other variables, or even use the groups as explanatory variables in other models. Specific approaches allow data to be weighted in cases of non-representativeness and missing data to be handled appropriately [25].

The LHC is currently one of the most effective methods for collecting retrospective data, such as that required for analyzing career trajectories within the HCWF. This method is now well-established in literature and enables the relatively rapid collection of detailed longitudinal information suitable for highly complex analyses. The use of an LHC can provide healthcare system stakeholders with valuable insights to support more effective management and future planning of the HCWF.
